# Un type rare de luxation d’épaule

**DOI:** 10.11604/pamj.2014.18.131.4536

**Published:** 2014-06-11

**Authors:** Issam Elouakili, Younes Ouchrif

**Affiliations:** 1 Service de Chirurgie Orthopédique, CHU, Rabat, Maroc

**Keywords:** Luxation, épaule, traumatisme, dislocation, shoulder, trauma

## Image en medicine

La luxation de l’épaule, est définie par une perte de contact totale et permanente de la tête humérale avec la cavité glénoïde de la scapula, se produisant au décours d'un traumatisme. C'est une des urgences en chirurgie orthopédique dans le sens où l'intervention doit intervenir rapidement. D'une part pour le risque de compression d’éléments nobles, mais aussi pour l'avenir de l'articulation (déformation articulaire, instabilité, arthrose). La luxation de l’épaule dans sa forme erecta est une variété rare, elle représente 0.5% de toutes les luxations de l’épaule. Peu de cas ont été signalés dans la littérature. Nous rapportons le cas d'un jeune homme de 20 ans qui s'est présenté aux urgences avec une attitude typique de la luxation erecta. Le traitement a consisté en une rééduction en traction directe dans l'axe du membre sous une légere sédation. L'immobilisation en position de rotation interne pendant 3 semaines, et une rééducation précoce sont très recommandées dans la littérature.

**Figure 1:**
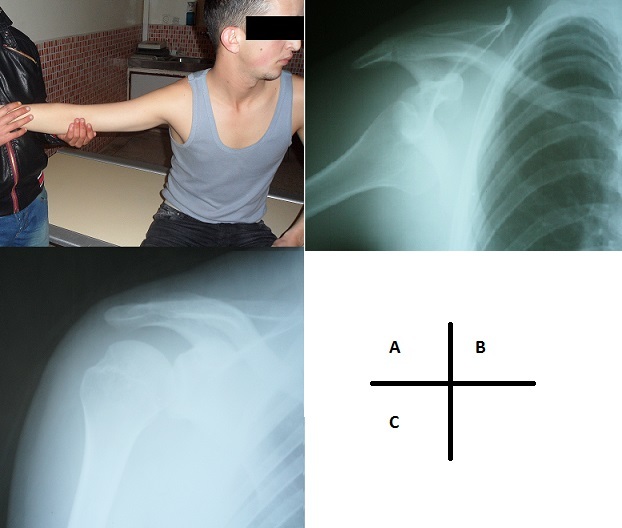
A) Position du patient aux urgences; B) Radiographie de l’épaule droite montrant une luxation erecta; C) Radiographie de contrôle après reduction

